# Identification of genes related to mental disorders by text mining

**DOI:** 10.1097/MD.0000000000017504

**Published:** 2019-10-18

**Authors:** Ying Wu, Meilin Dang, Hongxia Li, Xing Jin, Wenxiao Yang

**Affiliations:** a School of Humanities and Social Sciences, Shanxi Medical University,; b Affiliated Tumor Hospital, Shanxi Medical University, Taiyuan, China.

**Keywords:** gene, mental disorders, text mining

## Abstract

Mental disorders are important diseases with a high prevalence rate in the general population. Common mental disorders are complex diseases with high heritability, and their pathogenesis is the result of interactions between genetic and environmental factors. However, the relationship between mental disorders and genes is complex and difficult to evaluate. Additionally, some mental disorders involve numerous genes, and a single gene can also be associated with different types of mental disorders.

This study used text mining (including word frequency analysis, cluster analysis, and association analysis) of the PubMed database to identify genes related to mental disorders.

Word frequency analysis revealed 52 high-frequency genes important in studies of mental disorders. Cluster analysis showed that *5-HTT*, *SLC6A4*, and *MAOA* are common genetic factors in most mental disorders; the intra-group genes in each cluster were highly correlated. Some mental disorders may have common genetic factors; for example, there may be common genetic factors between ‘Affective Disorders’ and ‘Schizophrenia.’ Association analysis revealed 35 frequent itemsets and 25 association rules, indicating close associations among genes. The results of association rules showed that *CCK*, *MAOA*, and *5-HTT* are the most closely related.

We used text mining technology to analyze genes related to mental disorders to further summarize and clarify the relationships between mental disorders and genes as well as identify potential relationships, providing a foundation for future experiments. The results of the associative analysis also provide a reference for multi-gene studies of mental disorders.

## Abstract 2

Supplemental Digital Content is available in the text

## 1 Introduction

Mental disorders are important diseases with a high world-wide prevalence rate and characterized by high morbidity, high resource utilization, and high disability rates. From 1990 to 2010, the proportion of mental disorders in the global burden of disease increased by 37%,^[1]^ with 14% to 33% of adult disabilities related to mental illness. The prevalence of mental disorders in China increased from 3.2% to 7.2% in the mid-late 1970s to the current prevalence of 17.5%.^[2]^ Therefore, studies of the pathogenesis of mental disorders are urgently needed. Common mental disorders are complex diseases with high heritability, with schizophrenia, bipolar affective disorders, and major depression as typical polygenic genetic diseases.^[3]^ Their pathogenesis results from interactions between genetic and environmental factors.

In recent years, with the application of high-throughput sequencing technologies, recombinant DNA technology, and multidimensional nuclear magnetic resonance, molecular biology has rapidly evolved.^[4,5]^ Sequencing of the human genome and various patterns of biological genomes have produced large amounts of nucleic acid data.^[6,7]^ These genes can be divided into groups or categories and distinguished according to different phenotypes such as disease types or cell types,^[8]^ followed by experimental analysis to determine their relationships. However, the relationships between diseases and genetics are complex and difficult to evaluate. Schizophrenia is thought to be associated with the common genes, dopamine receptor gene,^[9]^ 5-hydroxytryptamine receptor gene, glutamate receptor gene, catechol-O-methyltransferase gene, and monoamine oxidase gene.^[10]^ Other candidate genes, such as Neuregulin 1 gene^[11]^ and myelin-associated glycoprotein gene^[12]^ are gradually being identified. Mood disorder is associated with the 5-hydroxytryptamine receptor gene and dopamine receptor gene.^[13]^ Additionally, some studies showed that the Reelin gene is associated with schizophrenia, bipolar disorder, and autism.^[14–16]^ A study from the Psychiatric Genomics Consortium found that the polygenic risk for bipolar affective disorder and the polygenic risk for schizophrenia were also associated with depression, and these disorders share some common genetic overlaps.^[17]^ Another study from the Cross-Disorder Group of the Psychiatric Genomics Consortium found that shared genetic variations exist between 5 types of mental disorders (bipolar affective disorder, autism, hyperactivity disorder, schizophrenia, and major depression).^[18]^ Thus, determining the relationships between diseases and genes as well as the interactions among genes related to mental disorders given these large amounts of genetic data is important for obtaining valuable information and identifying potential relationships.

Both biological data and biomedical literature are growing at extraordinary rates. The large body of literature contains important information that can be used for text mining.^[19]^ Text mining refers to the process of extracting latent and useful knowledge from text data to discover new information. Some researchers have extracted information about adverse drug reactions from descriptive texts on social media.^[20]^ Text mining methods were also used to explore medication regularity based on differentiation in traditional Chinese medicine for treating obesity.^[21]^ A study presented the application of text mining to assess and compare the interest in fitness tracking technology across eating disorder and health-related online communities.^[22]^ Various methods can be used for text mining, among which clustering refers to classifying data into different categories according to the similarity between the original data. Association analysis describes and determines whether symbiotic phenomena occur in existing data, mainly reflecting the relevance between factors to evaluate the possibility of events occurring together.

In this study, we used text mining technology to analyze genes related to mental disorders in the PubMed database. High-frequency genes related to mental disorders were identified and evaluated by cluster analysis and association analysis based on the co-occurrence relationship between mental disorders and genes. We explored the relationships between genes and mental disorders.

## 2 Methods

From PubMed (http://www.ncbi.nlm.nih.gov/PubMed), we retrieved 2562 relevant publications from September 1, 1966 to September 1, 2017 using the terms “Mental disorder {majr} AND genes {majr},” with no restriction on the language. The search results were saved in TXT format and used as the sample.

During sample processing with the MetaMap package in the National Library of Medicine, free terms were matched with the TXT format using Metathesaurus in the Unified Medical Language System. This method compares words in the abstract with concepts in Metathesaurus, and then uses an algorithm to identify matching words and assign each word a semantic type. The super word list in the Unified Medical Language System was divided into 134 semantic types.^[23]^ In this study, the semantic type concepts “mental or behavioral dysfunction” and “gene or genome” were statistically analyzed. We used the co-word method; in this method, if 2 concept words appeared together in the same sentence in the abstract, they were considered as related.

Terms extracted from the text were normalized and a wide range of gene concepts, such as “Genes” and “Allele” were deleted. Some concept terms were also excluded from being identified as gene names because they did not specifically refer to genes, such as ‘CSF,’ ‘LED,’ and ‘RAF’ in the literature searched. To highlight the key genes and facilitate analysis, we selected 52 genes with a frequency of more than 10 and analyzed these genes using the ‘wordcloud’ package. High-frequency genes and the 32 mental disorders related to these genes were used to construct a co-occurrence matrix in which the first column represented the name of the mental disorder and the first line represented the name of the gene. If a co-occurrence relationship was detected between the disorder and gene, it was marked as ‘1’, while a lack of correlation was marked as ‘0’ (see Table, Supplemental Content), which reflects the correlation between mental disorders and high-frequency genes. The co-occurrence matrix was analyzed using the ‘hclust’ package^[24]^ in R based on the types of mental disorders, and cluster analysis of high-frequency genes was conducted to further explore the relationship between genes and disorders. Finally, the ‘arules’ package^[25]^ of R was used to analyze the correlation of these high-frequency genes, explore the correlative strength among genes, and predict the regularity of gene occurrence.

Ethical approval was not necessary for this study, because all the data were obtained from the PubMed online database.

## 3 Results

### 3.1 Word frequency analysis

**Figure 1:**
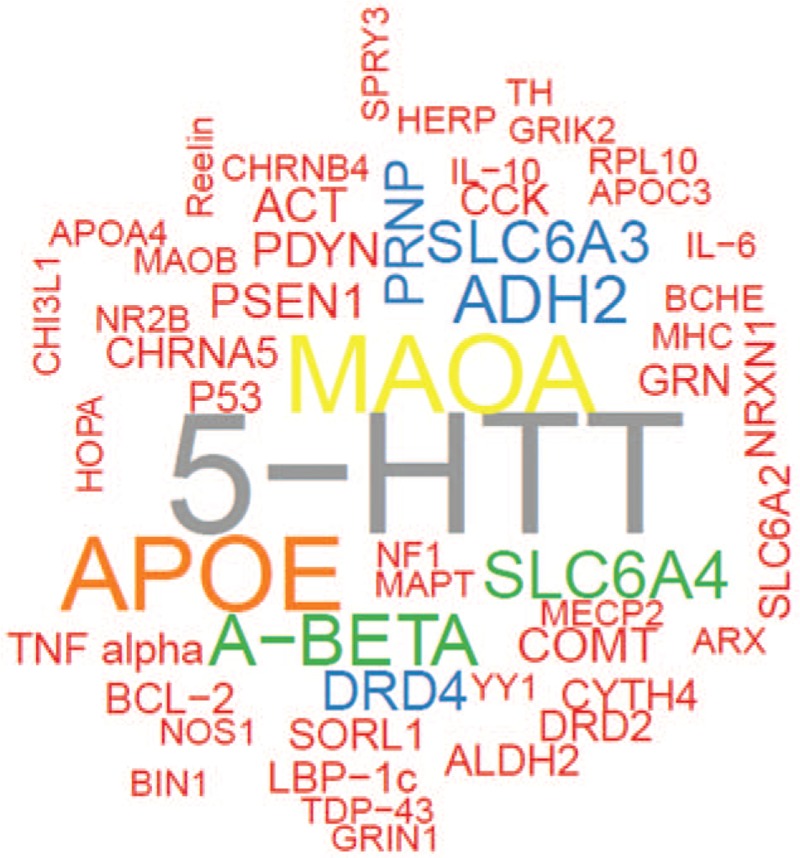
The word Cloud of 52 high frequency genes related to mental disorders.

Word frequency analysis is an important method for data mining, which refers to counting and analyzing the occurrence of important words in the text, and is a traditional and representative content analysis method in bibliometrics. The basic principle is to determine the hotspot and its changing trend by the frequency of words. In this study, the 52 high-frequency genes were arranged in descending order (Table 1) and the ‘wordcloud’ package in R was used to draw a word cloud map (Fig. 1), in which gene names were distinguished by different colors. A location closer to the center and a larger font size indicate a higher gene frequency. These high-frequency genes play important roles in studies of mental disorders.

**Table 1:**
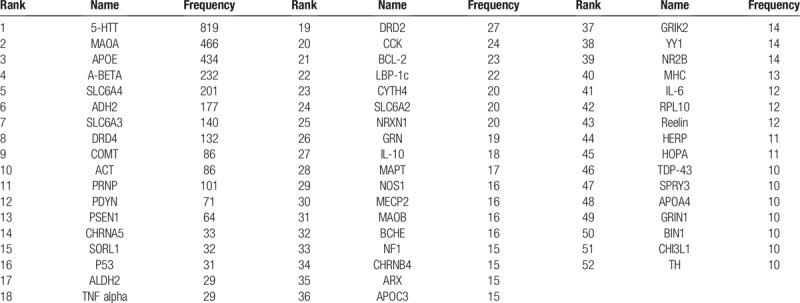
High frequency genes related to mental disorders.

### 3.2 Cluster analysis

**Figure 2:**
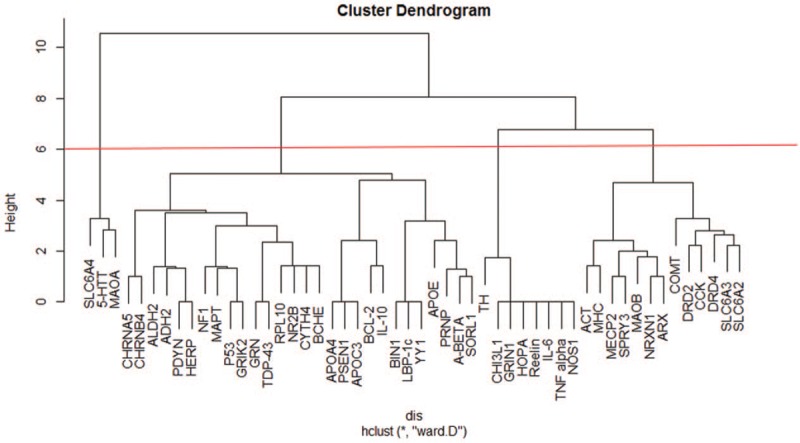
Cluster dendroram of 52 high frequency genes related to mental disorders.

After converting the format of the co-occurrence matrix of mental disorders and genes, a tree clustering graph was generated using the ‘hclust’ package in R (Fig. 2). In this figure, high-frequency genes were divided into 4 clusters. The first group included *5-HTT*, *SLC6A4*, and *MAOA*, which are related to 14 disorders, with each gene related to various disorders. These core genes were present at high frequencies. The second group included 28 genes related to 7 disorders, with most genes related to only one disorder. Notably, several key genes, such as *APOE* and *BCL-2*, were related to more than 3 disorders and linked the other genes together. The third group included 8 genes related to only 2 disorders. The fourth group included 13 genes, with key genes, such as *MOAB*, *DRD2*, *COMT*, *CCK*, *DRD4*, *SLC6A3*, and *SLC6A2* associated with other genes related to more than 3 disorders. The related disorders in each cluster may share similar genetic factors.

### 3.3 Association analysis

By using the ‘apriori’ package in the R program, we analyzed frequent itemsets and association rules of high-frequency itemsets and genes.

**Figure 3:**
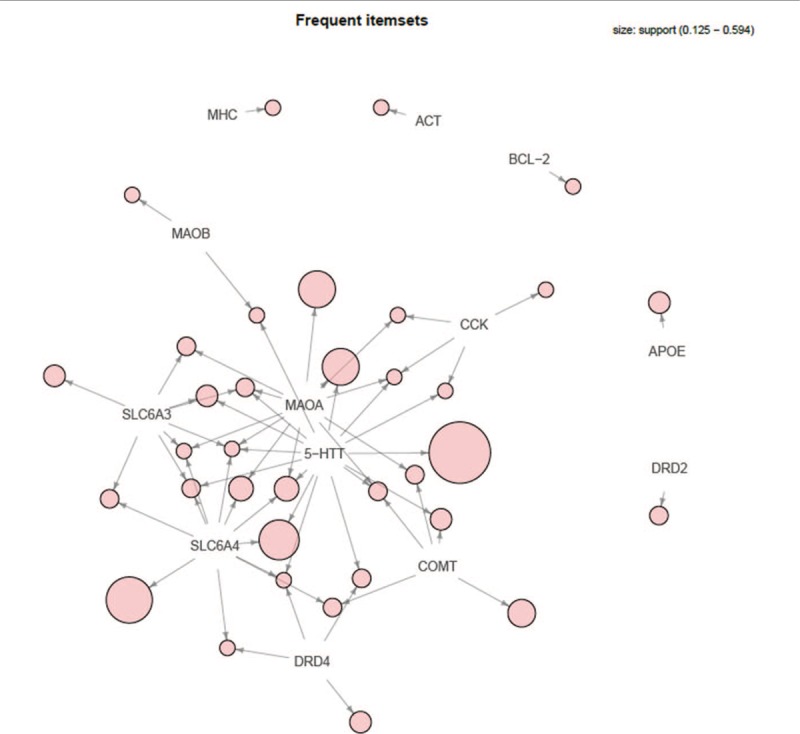
The visualization map of 35 frequent itemsets.

For frequent itemset analysis, mental disorders were treated as transactions and high-frequency genes as specific items. Under the default parameters (support 0.1, confidence 0.8), 35 frequent itemsets were obtained, among which there were 13 frequent 1-itemsets, 14 frequent 2-itemsets, 7 frequent 3-itemsets, and 1 frequent 4-itemsets (Fig. 3). The red circle represents ‘support,’ with a larger circle indicating greater ‘support.’ The ‘support’ of this case was 0.125 to 0.594. Each item is indicated as ‘support’ by a directed arrow, representing the corresponding item forming an itemset. Moreover, the parameter ‘target’ was set as ‘maximally frequent itemsets’ to obtain 2 frequent 2-itemsets: {*5-HTT*, *MAOB*} and {*SLC6A4*, *COMT*}; 3 frequent 3-itemsets: {*5-HTT*, *MAOA*, *CCK*}, {*5-HTT*, *SLC6A4*, *DRD4*}, and {*5-HTT*, *MAOA*, *COMT*}; and one frequent 4-itemsets {*5-HTT*, *MAOA*, *SLC6A4*, *SLC6A3*}. The genes in these frequent itemsets were closely related.

**Figure 4:**
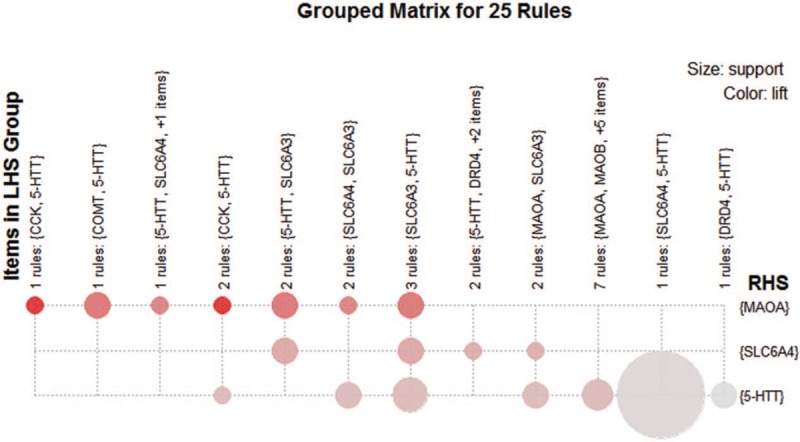
The visualization map of 25 association rules.

For association rule analysis, 25 association rules were obtained under default parameters (support 0.1, confidence 0.8) (Fig. 4). A larger circle indicated greater ‘support.’ A darker color indicated greater ‘lift.’ Rules with maximum ‘lift’ were (*CCK*) > (*MAOA*) and (*5-HTT*, *CCK*) > (*MAOA*), for which the ‘lift’ was 2.909. The rule with maximum ‘support’ was (*SLC6A4*) > (*5-HTT*), showing ‘support’ of 0.375. This result was used to predict the appearance rule of the genes.

## 4 Discussion

Various mental disorders are complex diseases involving multiple genes. Different mental disorders may be related to the same gene, and different genes may be related to the same disease. The relationships between mental disorders and genes are complex and difficult to evaluate. Previous studies have focused on the common genetic factors between 2 types of mental disorders,^[26,27]^ and some studies showed that multiple shared genetic factors exist among the 5 most common mental disorders.^[28]^ While numerous studies have been published containing potentially important information on these associations, diseases and gene laws have not been determined. In this study, a text mining method was used to analyze the relevant literature in the PubMed database.

First, 52 genes with the highest frequencies of being related to mental disorders were identified by frequency analysis, and a co-occurrence matrix of mental disorders and genes was constructed to determine the relationship between disorders and genes.

Second, these high-frequency genes were divided into 4 clusters by cluster analysis, with 3 core genes found to be related to various mental disorders and 9 key genes found to play important roles in connecting these genes. Fifty-two genes were clustered from small groups into larger groups according to distance. The similarity within clusters decreased gradually, and genes in the smallest groups were often associated with one or several mental disorders. Additionally, based on the clustering results, some mental disorders may have common genetic factors. The first cluster contained only 3 genes including *5-HTT*, *SLC6A4*, and *MAOA*, which were associated with 14 mental disorders. These results suggest that the 3 genes are common genetic factors in most mental disorders. The second and fourth clusters contained multiple genes, each of which was associated with only a few mental disorders and was linked by key genes. Intra-group genes in each cluster were highly correlated. For example, *CHRNA5* and *CHRNA4* in the second cluster were adjacent to each other and formed the smallest group, showing a close relationship with ‘Nicotine Dependence.’ Additionally, *APOC3*, *BCL-2*, and *IL-10* belonged to the second cluster. *BCL-2* was adjacent to *IL-10*, *BCL-2* was directly related to ‘Schizophrenia,’ ‘Dementia,’ and ‘Bipolar Disorder,’ *IL-10* was directly related to ‘Schizophrenia’ and ‘Alzheimer Disease,’ and *APOC3* was directly related to ‘Alzheimer Disease.’ Thus, ‘Schizophrenia,’ ‘Dementia,’ ‘Bipolar Disorder’, and ‘Alzheimer Disease’ may have common genetic factors, which should be confirmed by experimental analysis. The third cluster contained 8 genes related only to ‘Affective Disorders’ and ‘Schizophrenia’, suggesting that common genetic factors exist between the 2 mental disorders.

Finally, association analysis was conducted to evaluate the degree of associations among these high-frequency genes. Thirty-five frequent itemsets were obtained, which contained 2 maximally frequent 2-itemsets, 3 maximally frequent 3-itemsets, and 1 maximally frequent 4-itemsets. The genes in each frequent itemset were closely related to common mental disorders. For example, *5-HTT* and *MAOB* belonged to the 2 maximally 2-frequent itemsets, which were closely related to ‘Schizophrenia,’ ‘Autistic Disorder,’ and ‘Post-Traumatic Stress Disorder.’ Additionally, 25 association rules were obtained, indicating a close associative degree among genes. For example, 2 association rules, ‘(*CCK*) > (*MAOA*)’ and ‘(*5-HTT*, *CCK*) > (*MAOA*)’, with the largest degree of ‘lift’ suggested that *CCK*, *MAOA*, and *5-HTT* are closely related. These 3 genes were all related to ‘schizophrenia,’ ‘alcoholism,’ ‘mood disorders’, and ‘panic disorder,’ but ‘autistic disorder,’ ‘major depression,’ and ‘bipolar disorder’ were only related to *5-HTT* and *MAOA*. Thus, these mental disorders may also be related to *CCK*, which must be further analyzed.

There were some limitations to this study. To better match the MetaMap program and Metathesaurus, we only used the PubMed database as the source of literature data. In further research, we will evaluate additional medical professional databases. Additionally, there was a lack of negative detection due to the use of the co-word method for extracting concept words. When a term was stated in the same sentence of the abstract to be unrelated to a specific disease, the term was still considered to have co-occurred, impacting the results of the study.

In conclusion, this study used text mining technology to analyze genes related to mental disorders to further summarize and clarify the relationships between mental disorders and genes as well as identify potential relationships, providing a foundation for future experiments. The results of the associative analysis also provide a reference for multi-gene studies of mental disorders.

## Author contributions

**Conceptualization:** Ying Wu.

**Data curation:** Xing Jin.

**Formal analysis:** Hongxia Li.

**Funding acquisition:** Ying Wu.

**Investigation:** Hongxia Li.

**Methodology:** Xing Jin.

**Project administration:** Xing Jin.

**Resources:** Meilin Dang, Hongxia Li, Wenxiao Yang.

**Software:** Xing Jin, Wenxiao Yang.

**Validation:** Meilin Dang.

**Visualization:** Meilin Dang, Wenxiao Yang.

**Writing – original draft:** Ying Wu.

**Writing – review & editing:** Ying Wu.

## Supplementary Material

Supplemental Digital Content
